# Body Mass Index and COVID-19: An Overview Among an Italian Multicentric Cohort of Healthcare Workers in the Pre- and Post-Vaccination Eras—ORCHESTRA Project

**DOI:** 10.3390/vaccines13060660

**Published:** 2025-06-19

**Authors:** Gianluca Spiteri, Lorena Torroni, Maria Grazia Lourdes Monaco, Angela Carta, Francesco Taus, Alberto Modenese, Loretta Casolari, Maria Luisa Scapellato, Filippo Liviero, Francesca Larese Filon, Francesca Rui, Giuseppe Verlato, Stefano Porru

**Affiliations:** 1 Occupational Medicine Unit, University Hospital of Verona, 37134 Verona, Italy; gianluca.spiteri@aovr.veneto.it (G.S.); stefano.porru@univr.it (S.P.); 2 Departmental Faculty of Medicine, Saint Camillus International University of Health and Medical Sciences, 00131 Rome, Italy; 3 Unit of Epidemiology and Medical Statistics, Department of Diagnostics and Public Health, University of Verona, 37134 Verona, Italy; 4 Section of Occupational Medicine, Department of Diagnostics and Public Health, University of Verona, 37134 Verona, Italy; 5 Department of Biomedical, Metabolic and Neural Sciences, University of Modena and Reggio Emilia, 41125 Modena, Italy; 6 Health Surveillance and Workers Health promotion Service, University Hospital of Modena, 41125 Modena, Italy; 7 Department of Cardiac Thoracic Vascular Sciences and Public Health, University of Padova, 35128 Padua, Italy; 8 Occupational Medicine Unit, University Hospital of Padova, 35128 Padua, Italy; 9 Unit of Occupational Medicine, Department of Medical Science, University of Trieste, 34149 Trieste, Italy

**Keywords:** obesity, SARS-CoV-2, SARS-CoV-2 vaccination, serological response, risk factors, BMI

## Abstract

Background The prevalence of obesity is increasing all over the world, resulting in a global health emergency. The impact of obesity on the risk of SARS-CoV-2 infection and symptom severity, especially among high-risk working populations such as health workers, deserves further studies. Methods A multicentric retrospective cohort study was conducted among health workers at four Italian University Hospitals belonging to the ORCHESTRA Project. Data were collected through an online survey, investigating sociodemographic and clinical data, until September 2022. Results The questionnaire was filled out by 5777 health workers. The median age was 46 years old (I–III quartile 20–72) and 75.5% were females. Data on BMI was available for 5470 participants. Overweight and obese subjects amounted to 23.4% and 9.8%, respectively. Naïve health workers were the majority (57.4%). Overweight and obese subjects were at a higher risk of infection only before vaccination with respect to normoweight subjects (RRR = 1.28 (IC 95% 1.01–1.62, *p* = 0.039) and 1.36 (1.00–1.86, *p* = 0.047), respectively). Major acute and post-acute COVID-19 symptoms were more common among obese subjects, as compared to those with a normal weight (35.2% vs. 23.5%, and 14.2% vs. 9.3%). BMI did not reduce antibody levels after vaccination. On the contrary, overweight and obese health workers had a significantly higher RGM after the third dose (1.12 and 1.48, respectively; normal weight as reference). Conclusions Overweight and obese subjects are at a higher risk of SARS-CoV-2 infection. However, SARS-CoV-2 vaccination fosters a high antibody response even in these individuals. Vaccination against SARS-CoV-2 should be prioritized in subjects with a high BMI, especially in highly exposed workers, such as health workers.

## 1. Introduction

Obesity and overweight, defined as excessive fat deposition, are characterized by a pathological increase in body weight with respect to an individual’s height [1]. World Health Organization estimates for 2022 showed that about 2.5 billion adults (43% of the adult population worldwide) were overweight, and more than 890 million suffered from obesity (16% of the population aged 18 years and older) [1]. In Italy, data from the European Union National Health and Wellbeing Survey revealed that, in 2013, about 35 percent and 13 percent of the general adult population were overweight or obese, respectively [2]. More recently, Okunogbe et al. estimated the overall prevalence of overweight and obesity in 161 countries. Their results showed that, in Italy, the overall prevalence of overweight and obesity was 57.9% in 2019, with higher values in the adult male population (69.8%) [3]. These data show that the prevalence of obesity is a global health emergency.

Indeed, the scientific literature has shown that obese individuals have an increased risk of diabetes and cardiovascular, neurological, chronic respiratory, and cancer diseases [4]. Moreover, obesity has been correlated with a higher risk of getting infected and having a reduced humoral response to vaccinations. This effect appears to be related to the pro-inflammatory action of adipose tissue, resulting in chronic low-grade inflammation linked to an altered immune system and increased susceptibility to infection [5,6,7]. Several reviews have reported that obese individuals are more prone to post-surgical infections, skin infections, H1N1 influenza, and periodontal disease [8,9,10].

Furthermore, the humoral response against the most common pathogens after vaccination in obese subjects is also reduced. Antibody titers against influenza, hepatitis B, rabies, and tetanus showed significantly lower levels in obese people than in normal-weight subjects after vaccination [11]. After the emergence of the COVID-19 pandemic, several studies have evaluated the impact of obesity on the risk of SARS-CoV-2 infection and its severity, showing an increased risk of poor outcomes, invasive mechanical ventilation, mortality, and long COVID in obese subjects [5,7,12,13]. On the other hand, the effect of obesity on antibody response after vaccination for COVID-19 is still unclear. While some studies have shown that an increased BMI negatively impacts antibody levels, likewise, as do other comorbidities [14,15,16,17,18,19], and in other studies, such an inverse correlation is not significant or missing [6,20,21,22,23,24]. In the same way, the different impacts of BMI by gender are also unclear. Indeed, Yamamoto et al. reported significant differences between sexes, showing that a BMI over 30 was related to a lower antibody level only in men [25].

Most studies investigating these issues have been conducted on health workers (HWs) for several reasons. First, HWs were the occupational group at the greatest risk during the COVID-19 pandemic, representing an area of interest in evaluating the impact of occupational exposure. Second, most HWs underwent periodic antibody assays in the pre- and post-vaccination periods, providing data on antibody response trends over time. Finally, HWs were periodically screened by swab, so data on the incidence of SARS-CoV-2 infections, even asymptomatic ones, are more reliable than in the general population.

This study aimed to evaluate the impact of overweight and obesity within a multicentric population of HWs during the pre- and post-vaccination phases of the SARS-CoV-2 pandemic on the following:

- The risk of SARS-CoV-2 infection;
- The risk of post-acute COVID-19 symptoms;
- The antibody response.

## 2. Materials and Methods

### 2.1. Study Design and Setting

This study was addressed to HWs included in the Horizon 2020 ORCHESTRA Project (https://orchestra-cohort.eu/; accessed on 16 June 2025).

An online survey, which retrospectively investigated socio-demographic information and clinical data, including details on SARS-CoV-2 vaccination and previous infection, was sent to HWs employed at the University Hospitals of Modena (n = 610), Padua (n = 6728), Trieste (n = 6230), and Verona (n = 8183) in June 2022. A monthly reminder was sent to all HWs. Data were collected until September 2022.

The structure of the questionnaire and the details of the information collected have already been described in a previous ORCHESTRA study [26].

This study followed the “Strengthening the Reporting of Observational Studies in Epidemiology” (STROBE) reporting guidelines and was approved by the Italian Medicine Agency (AIFA) and the Ethics Committee of the Italian National Institute of Infectious Diseases (INMI) Lazzaro Spallanzani. The local ethical boards also approved the study.

### 2.2. BMI Categories

BMI was obtained using the following formula:

- Person’s weight in kilograms divided by the square of the person’s height in meters (kg/m^2^).

Each participant who reported data on height and weight was included in one of the four BMI categories (underweight, BMI < 18.5; normal weight, 18.5–24.9; overweight, 25–29.9; obesity, >30), in agreement with the WHO classification [27].

### 2.3. Vaccination Status

Based on the vaccination status of each HW, we defined infection periods based on vaccination status at the time of infection, distinguishing between the following:

- Pre-vaccination phase: before the administration of the first dose of the SARS-CoV-2 vaccine;
- Between the 1st and 3rd dose: after the administration of the 1st dose and before the 3rd dose;
- After 3rd dose: after administering the 3rd dose of the vaccine.

Case definition, symptom classification, and serological analyses were conducted.

A participant was categorized as infected if he/she reported at least one positive nasopharyngeal swab for SARS-CoV-2 until the questionnaire was filled out.

The severity of symptoms was classified as follows:

- No symptoms;
- Minor symptoms, if the infected HWs reported at least one symptom among fever, abdominal pain, vomiting and/or nausea, dysgeusia or ageusia, loss of smell, cough, chest pain and/or tightness, diarrhea, dyspnea, weight loss, fatigue and/or malaise, headache, muscle pain (myalgia) or joint pain (arthralgia), runny nose (rhinorrhea), sore throat, conjunctivitis, skin rash, dizziness, insomnia, rhinitis, concentration disorders, and chills;
- Major symptoms, if the infected HWs reported at least one symptom among altered consciousness and/or confusion, seizures, inability to walk, lymphadenopathy, bleeding, fainting (syncopal episodes), memory loss, anxiety, depression, anorexia, and aphasia.

Post-acute symptoms were defined as the persistence of at least one symptom after a negative swab.

In the Verona Cohort, serological response was assessed on the day of administering the 2nd dose, six months after the 2nd dose, and 1 month after the 3rd dose. Samples were analyzed with the Liaison SARS-CoV-2 TrimericS IgG test (Diasorin), a chemiluminescence immunoassay (CLIA), which quantitatively detected antitrimeric spike protein-specific IgG antibodies. The test results are reported as BAU/mL (binding antibody unit per mL). Samples that exceeded the linearity range underwent a 1:20 dilution.

### 2.4. Statistical Analysis

The normality of continuous variables was assessed using the Shapiro–Wilk test. Variables that deviated significantly from normality were analyzed using non-parametric methods or were appropriately transformed.

Since the data were skewed, quantitative variables are summarized as the median and interquartile range (I–III quartile), and categorical variables are frequencies and percentages. Where appropriate, the significance of differences among BMI groups was evaluated using Kruskal–Wallis or Fisher’s exact tests for continuous or categorical variables.

We evaluated the risk factors between infection periods (before vaccination, between the 1st and 3rd dose, and after the 3rd dose) using multinomial logistic regression models, controlling for sex, age (by ten-year increase), BMI categories (underweight, normoweight, overweight, and obese), comorbidities, and job title (administrative, nurse, technician, physician, and other HW). Each outcome’s relative risk ratio (RRR) with a 95% confidence interval (CI) is reported.

We used regression models to investigate the humoral response after each vaccine dose (humoral response after 1st, 2nd, and 3rd doses of vaccines). The serum concentration of each test was log-transformed to fulfill the assumption of normality. Association estimates are expressed as ratios of geometric means (RGMs) with a 95% CI. The regression models were adjusted for sex, age, BMI, previous infections, and comorbidity. Lag time, expressed as the elapsed months between second-dose administration and sampling, was also used to adjust the model for the II serum test.

Participants with missing BMI data were excluded from the corresponding analyses. For all other variables, missing data were not imputed, and analyses were performed using available-case (complete-case) methods.

The level of statistical significance was set at 5%. The analysis used STATA software, version 18.0 (StataCorp, College Station, TX, USA).

## 3. Results

A total of 5777 HWs filled in the questionnaire. The response rate was 26,6% among the whole study population (41.8% in Modena, 39.5% in Verona, 17.9% in Padua, and 17.4% in Trieste). Analyses were performed on 5470 HWs for whom data on BMI were available. The number of HWs included in the analyses for each cohort is specified in Table 1.

The median age of the participants was 46 (IQR 20–72) and they were mainly female (75.5%), in line with the composition of the national HW workforce (Table 1). As for BMI, the participants mostly had a normal weight (62.7%), while overweight and obese subjects amounted to 23.4% and 9.8%, respectively.

The majority of the HWs had not been previously infected (57.4%). On the other hand, 38.2% of the subjects were infected before vaccination or after the third dose (11.0% and 27.2%, respectively). Only a few, 3%, reported a SARS-CoV-2 infection between the first and the third dose of the vaccine. The socio-demographic and clinical characteristics of the study population are shown in Table 1.

The cumulative infection incidence and the percentage of infection by vaccination status were significantly affected by BMI category (*p* = 0.014). Obese HWs had a higher incidence of SARS-CoV-2 infection before vaccination (BV) with respect to those with a normal weight (14.5% vs. 11.0%), while a lower incidence was observed between first and third dose and after third dose. The distribution of infection by vaccination status and BMI is described in Table 2.

The multinomial logistic regression model shows that age and job title were determinants of risk of infection (RI) both before the primary vaccination course and after the third dose. Indeed, the medium age categories (30–39 and 40–49 yo) had an RRR of 1.53 (CI 95% 1.08–2.17, *p* = 0.017) and 1.52 (1.06–2.17, *p* = 0.023) BV, respectively, while the older-aged HWs (50–59 and ≥60 yo) had a significantly lower RI after the third dose. Regarding job title, nurses had a higher RI BV and after the third dose (RRR = 2.04, 1.39–2.99; *p* < 0.001 and 1.49, 1.14–1.94; *p* = 0.003), while physicians and technicians had a higher RI only after the third dose (RRR = 1.44, 1.08–1.91; *p* = 0.011 and 1.39, 1.00–1.94; *p* = 0.049 respectively). On the other hand, BMI and comorbidities influenced RI only BV (RRR = 1.28,1.01–1.62; *p* = 0.039 and 1.36, 1.00–1.86; *p* = 0.047) in overweight and obese HWs, respectively; RRR = 1.44 (1.15–1.82; *p* = 0.002) in HWs who suffered from at least a comorbidity) (Table 3).

Considering the severity of infection, there was a significant association between acute symptoms and BMI (*p* = 0.013), with normal-weight and underweight subjects presenting mainly minor symptoms (61.1% and 66.7%, respectively). In contrast, obese subjects had the highest prevalence of severe symptoms (35.2%) (Table 4). Moreover, post-acute symptoms, both mild and severe, were more common among obese HWs, as compared to those with a normal weight (47.3% and 14.2% vs. 36.7% and 9.3%). On the other hand, no relevant differences were detected between overweight and normal-weight HWs (Table 4).

Linear regression on the humoral response after the first, second, and third doses showed that the main determinants of antibody levels were age class, previous infections, comorbidities, and the time elapsed between the second dose and the sampling (Table 5). The effects of each factor changed during the vaccination course. Indeed, older age was negatively related to RGM after either the first or second dose, while values were higher after the third dose in the 50–59 and >60 years old categories. As expected, previously infected HWs had a higher RGM after the first and second doses. On the contrary, administering the third dose was related to a slightly lower RGM in these subjects. Comorbidity had a negative influence on RGM after the first and second doses, too. No significant differences were reported after the third dose. Sex and BMI did not have a significant impact on RGM. In particular, RGM did not differ among BMI categories after the first and second doses, while overweight and obese HWs had a higher RGM after the third dose, using normoweight as reference (1.12 and 1.48, respectively, Table 5).

## 4. Discussion

The prevalence of overweight and obesity in our study population was 33.2%. This data is significantly lower than the percentage that Okunogbe reported for the general Italian population in 2022 (57.9%) [3]. This difference could be explained by the fact that overweight and obesity increase with age, especially in the retired population, reaching 67.9% in subjects aged 65 or older [28]. For the same reason, the lowest prevalence was reported in the Modena cohort, in which only residents were included (median age of 30 years, IQR 20–65). Another cause of the lower prevalence in HWs is probably related to the “healthy worker effect”, a process of excluding unhealthy individuals, leading to a health status difference between the workforce and the general population [29].

The overall percentage of HWs previously infected up to July 2022 was 43.1%, with an incidence that waved significantly during the different phases of the pandemic. As previously reported in the Orchestra HW cohorts, SARS-CoV-2 infection incidence collapsed after the beginning of the vaccination campaign and increased dramatically after the Omicron variant surge [30]. The lowest percentage of infection was among underweight subjects (39.8%). This data aligns with the findings of a study involving 5897 Italian HWs. Indeed, during the study period, from March 2020 to January 2021, the cumulative incidence of SARS-CoV-2 infection was lower in underweight HWs (8.3%) as compared to other BMI classes (12.2%, 14.8%, and 17.6% in normal-weight, overweight, and obese HWs, respectively). However, the difference was not significant in the multiple logistic regression analysis [31]. On the other hand, a review by Dobner et al. showed that the RI in general, not only of the respiratory tract, had a U-shaped relation to BMI, with normal-weight subjects at a lower risk relative to both underweight and obese individuals [10]. This difference confirms the specificity of COVID-19 risk factors, warranting an in-depth analysis of the particular risk factors of this disease.

In contrast, a higher risk was reported in overweight and obese HWs, even if only in the pre-vaccination pandemic phase. Indeed, the cumulative incidence of the entire study period was similar in these BMI categories to the normal-weight class (46.6% and 45.1% vs. 45.8%), while the incidence BV showed a positive trend corresponding with an increase in BMI (11.0%, 12.6%, and 14.5% in normoweight, overweight, and obese, respectively). This effect was qlwo evident in the multinomial logistic regression, in which overweight and obese had an RRR of 1.28 and 1.36, with respect to normoweight. At the same time, no significant differences in RRR were reported after vaccination. The same pattern was seen in HWs suffering from other comorbidities (RRR = 1.44 in the pre-vaccination phase, not significant after administering two or three vaccine doses).

A comparison with the literature data is challenging, because only a few studies have investigated the role of BMI in the risk of SARS-CoV-2 infection among HWs, focusing only on sex, age, job category, and the use of Personal Protective Equipment. In the previously mentioned article by Modenese et al., a higher OR in overweight and obese HWs was reported during the pre-vaccination period. Moreover, their ORs were very similar to our results (1.27 vs. 1.28 and 1.38 vs. 1.36 in overweight and obese, respectively) and increased with an increasing BMI category, confirming the trend shown by our data [31]. A higher risk of SARS-CoV-2 infection in obese HWs was also reported in the study by Rizza et al., evaluating the incidence during the first months of the pandemic (from March to May 2020). The OR was 7.15 in subjects with a BMI of > 30, compared to the BMI < 25 category. The stronger association with BMI reported in this research could be related to the different reference groups (BMI < 25, including underweight), the short follow-up, and the very low number of infections [32]. Studies on the general population have confirmed a higher RI in obese subjects. A meta-analysis by Popkin et al., including 20 studies up to June 2020, found an OR of 1.46 for infection in this category [33].

Even more scant data on the correlation between BMI and RI during the post-vaccination period are available. A comparison of the RI among BMI categories in the post-vaccination phase (from December 2020 to May 2021) was carried out by Alishaq et al. They used a Cox regression model, showing that HWs with a BMI of < 30 had an HR significantly higher than obese HWs (HR = 2.99) [34]. Furthermore, no influence of BMI on the RI was found among 267 HWs during the Omicron period (February–March 2022) in a study that assessed SARS-CoV-2 anti-N antibodies [35]. These findings highlight vaccination’s key role and effectiveness in preventing SARS-CoV-2 infection, especially in the most vulnerable categories.

Regarding COVID-19 severity related to BMI class, we found no significant difference in the percentage of symptomatic or asymptomatic infections. On the other hand, data on symptom severity showed that among obese HWs, there was a higher percentage of subjects who reported major symptoms as compared to other categories, including overweight. Moreover, post-acute symptom persistence was the highest in obese HWs (61.4% vs. 57.5%, 35.7%, and 50.2%, in underweight, normal-weight, and overweight, respectively). These data are in line with the scientific literature, which shows an increased severity of SARS-CoV-2 infection and a higher incidence of long COVID in obese subjects. Indeed, data reported in systematic reviews and meta-analyses confirm that individuals with a BMI of > 30 have a higher risk of being hospitalized and a higher mortality rate. The association appeared stronger in higher BMI categories (second and third obesity grades) [5,7,12]. Moreover, data from an Italian study by Vimercati et al. showed that the BMI mean was higher in subjects who experienced long COVID, as compared to individuals who had no symptoms after COVID-19 infection, and the OR of suffering from long COVID in overweight participants was 1.6 (with reference to normal-weight subjects) [13]. Interestingly, our data showed that this effect waned during the pandemic. Indeed, the pre-vaccination and post-third-dose periods analysis reported that overweight and obese HWs had a higher incidence of major symptoms in the first phase (results not statistically significant). On the other hand, the risk of acute and post-acute major symptoms was lower in overweight and only slightly higher in obese HWs after third-dose administration, as compared to normal-weight HWs, confirming that vaccination dramatically reduced the effect of obesity on the risk of developing severe symptoms, as well as of being infected (Supplementary Table S1).

Finally, we evaluated the humoral response after each dose of vaccine (first, second, and booster dose). We found that the main determinants of SARS-CoV-2 antibody levels were age, previous infection, comorbidities, and the time elapsed since the last dose. Indeed, previously infected HWs had a higher anti-S titer after the first and second dose (RMG = 10.60 and 2.83, respectively), while an older age, comorbidities, and the number of months after the last dose were inversely related to the median antibody levels. These results are consistent with the findings reported in previous papers involving larger ORCHESTRA HW populations. Visci et al. investigated the serological response among 17,257 HWs from the Bologna, Brescia, and Verona cohorts from 21 days to 3 months after the first dose. They found that a 10-year increase was related to a decreased quantitative response (RR = 0.83) and time since vaccination per 14-day interval (RR = 0.97). Previously infected HWs had higher SARS-CoV-2 anti-S IgG (RR = 4.39), with a lower increase than that shown by other results related to the first dose. This difference can be explained by the fact that the previous paper included samples collected after the first and second doses [36]. Similar trends were reported in another ORCHESTRA study carried out among 60,000 HWs from eight European cohorts six months after the first dose. This paper also confirmed that the booster effect of a previous infection diminished with time (RR in previously infected = 2.26) [37]. Moreover, in agreement with our findings, a previous ORCHESTRA Project study involving 2941 HWs from seven European centers reported a negative effect of comorbidities on antibody levels, showing an RR = 0.87 in HWs suffering from hypertension and an RR = 0.89 in participants with at least two comorbidities [19].

More controversial are the results regarding the influence of BMI on humoral response. Indeed, we did not find any differences in the geometric mean values in overweight and obese HWs compared with those with a normal weight, except for a significant increase in obesity after the third dose. However, there is no consensus on this topic in the available literature. Watanabe et al. investigated a small group of HWs (86), and they did not find any correlation between BMI and serological response 1–4 weeks after the second dose [24]. Moreover, a study involving 290 Greek HWs reported slightly reduced antibody levels in obese participants one and six months after the second dose, but these results did not reach significance [23]. Furthermore, Golec et al. reported that BMI did not affect antibody levels 8 months after the second dose among a population of 242 HWs [14]. On the other hand, a subsequent study on the same population found a weak positive correlation 21 days after the booster dose (r = 0.22) [20]. In contrast, a lower antibody geometric mean concentration was shown in overweight and obese HW cohorts by Pellini et al., as compared to underweight and normal-weight subjects [11]. Interestingly, a study on 2435 HWs found a reduction in anti-S IgG 2 months after the second dose only in men, while no significant differences were reported in women. The lack of homogeneity in correlations across studies and data on the possible role of BMI only in men seems to support what Golec et al. hypothesized about the limited role of BMI in being able to identify subjects with a reduced humoral response. Indeed, considering that the impairment of the immune response is mainly related to the chronic pro-inflammatory role of abdominal fat, BMI should not be the only parameter to assess the risk of decreased immune response, particularly among women.

Our study has some limitations. BMI is the simplest and the most used method to classify large populations and determine health issues regarding overweight and obese subjects. However, it does not consider adipose tissue distribution and the amount of visceral adipose tissue, which is related to the pro-inflammatory effect [25]. All data were collected through a self-administered questionnaire. This aspect may have resulted in a misdating of previous infections and vaccinations, as well as an incorrect determination of symptom classification and of BMI in some participants. Moreover, questionnaire completion was on a voluntary basis, so the study population cannot be considered as representative of the entire working population of the cohorts involved. Furthermore, only Italian centers were included in the study due to the availability of BMI data. Therefore, ethnicity reflects the composition of the study population and limits the generalizability of the findings across Europe.

Finally, the unbalanced sex distribution limited sex-stratified analyses. Therefore, potential sex-specific differences in the relationship between BMI and immune response could not be thoroughly explored.

However, the high number of HWs included in the analyses and the multicentric design of the research ensure the reliability of our results. Furthermore, to the best of our knowledge, this is the only study on HWs that evaluated all pandemic phases. This lets us compare the influence of different BMI classes in the different periods of the pandemic. In addition, this is the only research that has investigated multiple effects of BMI on COVID-19 among HWs, including the RI, symptom severity, and humoral response to vaccination.

## 5. Conclusions

Our data showed that overweight and obese HWs were at a higher risk of SARS-CoV-2 infection and severe symptoms BV. However, the administration of a primary course overrides this increase.

The humoral response did not vary among BMI classes after the primary course and the booster dose, confirming that overweight and obesity alone did not affect the effectiveness of the vaccination.

These results confirm the urgency and effectiveness of vaccination administration in overweight and obese subjects, suggesting that public health policies should also consider BMI for prioritizing the primary-course vaccine. On the other hand, after administering a primary course of the vaccine, it seems that no additional doses should be provided to obese people who are not affected by other comorbidities beyond those recommended for a specific professional or age category.

Further studies that include body composition analyses and data on inflammatory markers should be carried out on the correlation of overweight and obesity with immunity to better understand the biological mechanisms of the observed effects.

## Figures and Tables

**Table 1:** Sociodemographic and clinical characteristics of the study population.

		Centers			
	Total	MO	PD	TS	VR
	(n = 5470)	(n = 231)	(n = 1165)	(n = 1006)	(n = 3068)
**Sex**					
Male	1310 (24.0%)	93 (40.3%)	215 (18.5%)	270 (26.8%)	732 (24.0%)
Female	4131 (75.5%)	138 (59.7%)	946 (81.2%)	732 (72.8%)	2315 (75.8%)
Other	5 (0.1%)	0 (0.0%)	2 (0.2%)	1 (0.1%)	2 (0.1%)
Not answered	9 (0.1%)	0 (0.0%)	1 (0.1%)	3 (0.3%)	5 (0.2%)
Missing	15 (0.3%)	0 (0.0%)	1 (0.1%)	0 (0.0%)	14 (0.5%)
Age *	46 (20–72)	30 (20–65)	50 (23–70)	47 (24–67)	46 (20–72)
BMI *	23 (14–63)	22 (17–50)	23 (14–53)	24 (16–63)	23 (15–58)
Underweight	223 (4.1%)	9 (3.9%)	49 (4.2%)	39 (3.9%)	126 (4.1%)
Normal weight	3428 (62.7%)	175 (75.8%)	687 (59.0%)	563 (56.0%)	2003 (65.3%)
Overweight	1282 (23.4%)	38 (16.5%)	296 (25.4%)	289 (28.7%)	659 (21.5%)
Obese	537 (9.8%)	9 (3.9%)	133 (11.4%)	115 (11.4%)	280 (9.1%)
**Country of birth**					
Italy	5232 (95.7%)	221 (95.7%)	1125 (96.6%)	947 (94.1%)	2939 (95.8%)
Other EU	102 (1.9%)	4 (1.7%)	22 (1.9%)	20 (2.0%)	56 (1.8%)
ExtraEU	108 (2.0%)	5 (2.2%)	18 (1.5%)	34 (3.4%)	51 (1.7%)
Not answered	7 (0.1%)	0 (0.0%)	0 (0.0%)	3 (0.3%)	4 (0.1%)
Missing	21 (0.3%)	1 (0.4%)	0 (0.0%)	2 (0.2%)	18 (0.6%)
**Ethnicity**					
Caucasian	4329 (79.1%)	218 (94.4%)	823 (70.6%)	795 (79.0%)	2493 (81.3%)
Other	532 (9.7%)	10 (4.3%)	152 (13.0%)	106 (10.5%)	264 (8.6%)
Not answered	383 (7.0%)	2 (0.9%)	125 (10.7%)	54 (5.4%)	202 (6.6%)
Missing	226 (4.2%)	1 (0.4%)	65 (5.6%)	51 (5.1%)	109 (3.6%)
**Job**					
Administrative	498 (9.1%)	0 (0.0%)	90 (7.7%)	82 (8.2%)	326 (10.6%)
Nurse	1851 (33.8%)	1 (0.4%)	563 (48.3%)	346 (34.4%)	941 (30.7%)
Technician	409 (7.5%)	0 (0.0%)	84 (7.2%)	76 (7.6%)	249 (8.1%)
Physicians	1190 (21.7%)	221 (95.7%)	210 (18.0%)	194 (19.3%)	565 (18.4%)
Other	1387 (25.4%)	5 (2.2%)	216 (18.5%)	280 (27.8%)	886 (28.9%)
Missing	135 (2.5%)	4 (1.7%)	2 (0.2%)	28 (2.8%)	101 (3.3%)
**Infection period**					
Not infected	2967 (54.2%)	101 (49.3%)	543 (48.5%)	605 (65.8%)	1718 (57.7%)
Before vaccination	601 (11.0%)	20 (9.8%)	133 (11.9%)	91 (9.9%)	357 (12.0%)
Between 1st and 3rd dose	165 (3.0%)	7 (3.4%)	58 (5.2%)	29 (3.2%)	71 (2.4%)
After 3rd dose	1485 (27.2%)	77 (37.6%)	385 (34.4%)	194 (21.1%)	829 (27.9%)
Missing	252 (4.6%)	26 (11.3%)	46 (3.9%)	87 (8.6%)	93 (3.0%)

* Median, I–III quartile.

**Table 2:** SARS-CoV-2 infection by vaccination status at time of infection and BMI.

		BMI				
	Total	Normoweight	Underweight	Overweight	Obese	*p*-Value
	(n = 5470)	(n = 3428)	(n = 223)	(n = 1282)	(n = 537)	
**Infection by vaccination status**						
Not infected	2967 (54.2%)	1858 (54.2%)	130 (58.3%)	684 (53.4%)	295 (54.9%)	0.014
Before vaccination	601 (11.0%)	339 (9.9%)	22 (9.9%)	162 (12.6%)	78 (14.5%)	
Between 1st and 3rd dose	165 (3.0%)	112 (3.3%)	8 (3.6%)	30 (2.3%)	15 (2.8%)	
After 3rd dose	1485 (27.2%)	958 (27.9%)	56 (25.1%)	344 (26.8%)	127 (23.6%)	
Missing	252 (4.6%)	161 (4.7%)	7 (3.1%)	62 (4.8%)	22 (4.1%)	

**Table 3:** Multinomial logistic regression: relative risk ratio (RRR) among infection by vaccination status.

Infection by Vaccination Status			RRR (95% CI)	*p*-Value
No Infection			*Ref.*	
**Before vaccination**	**Age (years)**	20–29	*Ref.*	
		30–39	1.53 (1.08–2.17)	0.017
		40–49	1.52 (1.06–2.17)	0.023
		50–59	0.99 (0.71–1.41)	0.997
		≥60	0.73 (0.46–1.17)	0.192
	**Sex**	Male	*Ref.*	
		Female	1.15 (0.89–1.47)	0.266
	**BMI**	Normoweight	*Ref.*	
		Underweight	0.97 (0.59–1.61)	0.920
		Overweight	1.28 (1.01–1.62)	0.039
		Obese	1.36 (1.00–1.86)	0.047
	**Job**	Administrative	*Ref.*	
		Nurse	2.04 (1.39–2.99)	<0.001
		Technician	0.83 (0.48–1.44)	0.514
		Physician	1.30 (0.85–1.98)	0.223
		Other	1.15 (0.77–1.73)	0.481
	**Comorbidity**	No	*Ref.*	
		Yes	1.44 (1.15–1.82)	0.002
**Between 1st and 3rd dose**	**Age**	20–29	*Ref.*	
		30–39	1.44 (0.80–2.59)	0.219
		40–49	1.61 (0.88–2.92)	0.117
		50–59	0.96 (0.53–1.72)	0.896
		≥60	0.56 (0.23–1.39)	0.209
	**Sex**	Male	*Ref.*	
		Female	1.03 (0.67–1.59)	0.886
	**BMI**	Normoweight	*Ref.*	
		Underweight	1.15 (0.54–2.44)	0.717
		Overweight	0.83 (0.53–1.30)	0.427
		Obese	0.75 (0.40–1.41)	0.381
	**Job**	Administrative	*Ref.*	
		Nurse	1.82 (0.93–3.53)	0.078
		Technician	1.14 (0.48–7.71)	0.755
		Physician	1.09 (0.52–2.29)	0.805
		Other	0.88 (0.42–1.82)	0.742
	**Comorbidity**	Yes (No *Ref.*)	0.98 (0.63–1.52)	0.946
**After 3rd dose**	**Age**	20–29	*Ref.*	
		30–39	0.96 (0.76–1.20)	0.739
		40–49	1.18 (0.93–1.48)	0.163
		50–59	0.69 (0.55–0.86)	0.001
		≥60	0.59 (0.44–0.81)	0.001
	**Sex**	Male	*Ref.*	
		Female	1.12 (0.95–1.33)	0.178
	**BMI**	Normoweight	*Ref.*	
		Underweight	0.83 (0.58–1.18)	0.311
		Overweight	1.07 (0.90–1.27)	0.416
		Obese	0.86 (0.67–1.10)	0.240
	**Job**	Administrative	*Ref.*	
		Nurse	1.49 (1.14–1.94)	0.003
		Technician	1.39 (1.00–1.94)	0.049
		Physician	1.44 (1.08–1.91)	0.011
		Other	0.97 (0.74–1.28)	0.864
	**Comorbidity**	No	*Ref.*	
		Yes	1.00 (0.83–1.19)	0.993

**Table 4:** Association between acute and post-acute symptoms severity by BMI categories in infected HWs only.

		BMI				
	Total (n = 1941)	Normoweight (n = 1261)	Underweight (n = 78)	Overweight (n = 423)	Obese (n = 179)	p-Value *
Acute symptoms severity *						
No symptoms	286 (14.7%)	194 (15.4%)	5 (6.4%)	65 (15.4%)	22 (12.3%)	0.013
Minor symptoms	1171 (60.3%)	771 (61.1%)	52 (66.7%)	254 (60.0%)	94 (52.5%)	
Major symptoms	484 (25.0%)	296 (23.5%)	21 (26.9%)	104 (24.6%)	63 (35.2%)	
Post-acute severity symptoms *						
No symptoms	949 (51.3%)	649 (54.0%)	31 (40.3%)	204 (50.6%)	65 (38.5%)	0.003
Minor symptoms	719 (38.8%)	441 (36.7%)	38 (49.4%)	160 (39.7%)	80 (47.3%)	
Major symptoms	183 (9.9%)	112 (9.3%)	8 (10.4%)	39 (9.7%)	24 (14.2%)	

* Percentage and *p*-value were computed on available data.

**Table 5:** Linear regression models: humoral response after 1st, 2nd, and 3rd doses of vaccine by sociodemographic and clinical characteristics.

	First Serum Test *	*p-Value*	Second Serum Test *	*p-Value*	Third Serum Test *	*p-Value*
	RGM (95% IC) (n = 1586)		RGM (95% IC) (n = 2032)		RGM (95% IC) (n = 1655)	
**Sex**						
Male	*Ref.*					
Female	1.03 (0.87–1.22)	0.729	1.07 (0.96–1.19)	0.195	0.96 (0.86–1.06)	0.414
**Age**						
20–29	*Ref.*					
30–39	1.04 (0.77–1.41)	0.794	0.93 (0.77–1.12)	0.443	1.06 (0.89–1.27)	0.507
40–49	0.74 (0.55–0.99)	0.046	0.69 (0.57–0.83)	<0.001	1.08 (0.91–1.29)	0.362
50–59	0.52 (0.39–0.69)	<0.001	0.77 (0.65–0.92)	0.005	1.24 (1.05–1.47)	0.010
≥60	0.40 (0.29–0.56)	<0.001	0.65 (0.52–0.80)	<0.001	1.20 (0.99–1.46)	0.065
**BMI**						
Normoweight	*Ref.*					
Underweight	0.88 (0.60–1.29)	0.511	1.00 (0.79–1.28)	0.980	1.06 (0.88–1.33)	0.623
Overweight	1.08 (0.91–1.28)	0.388	1.02 (0.92–1.13)	0.687	1.12 (1.01–1.23)	0.032
Obese	0.88 (0.70–1.11)	0.293	1.00 (0.87–1.15)	0.991	1.48 (1.29–1.71)	<0.001
**Previous infection**						
No	*Ref.*					
Yes	10.60 (8.60–13.07)	<0.001	2.83 (2.50–3.22)	0.017	0.88 (0.79–0.98)	0.019
**Comorbidity**						
No	*Ref.*					
Yes	0.79 (0.66–0.93)	0.005	0.88 (0.79–0.98)	0.017	0.97 (0.88–1.08)	0.611
**Lag time** (months)			0.79 (0.74–0.85)	<0.001		

* Humoral response after each vaccine dose (1st, 2nd, and 3rd dose of vaccine). The ratios of Geometric Means (RGMs) were obtained by exponentiating multiple linear regression coefficients of the log-serum test. All regression models were adjusted for sex, age, BMI, previous infections, and comorbidity. Lag time was also used to adjust the II serum test model.

## Data Availability

The datasets generated during the current study are not publicly available because they contain sensitive data to be treated under data protection laws and regulations. Appropriate forms of data sharing can be arranged after a reasonable request to the first author.

## Supplementary Materials

The following supporting information can be downloaded at: https://www.mdpi.com/article/10.3390/vaccines13060660/s1, Supplementary Table S1. Acute and post-acute symptom severity by BMI category.

## References

[1] Obesity and Overweight. https://www.who.int/news-room/fact-sheets/detail/obesity-and-overweight.

[2] DiBonaventura M., Nicolucci A., Meincke H., Le Lay A., Fournier J. (2018). Obesity in Germany and Italy: Prevalence, comorbidities, and associations with patient outcomes. Clin. Outcomes Res..

[3] Okunogbe A., Nugent R., Spencer G., Powis J., Ralston J., Wilding J. (2022). Economic impacts of overweight and obesity: Current and future estimates for 161 countries. BMJ Glob. Health.

[4] Global Burden and Strength of Evidence for 88 Risk Factors in 204 Countries and 811 Subnational Locations, 1990–2021: A Systematic Analysis for the Global Burden of Disease Study 2021. https://pubmed.ncbi.nlm.nih.gov/38762324/.

[5] Aghili S.M.M., Ebrahimpur M., Arjmand B., Shadman Z., Pejman Sani M., Qorbani M., Larijani B., Payab M. (2021). Obesity in COVID-19 era, implications for mechanisms, comorbidities, and prognosis: A review and meta-analysis. Int. J. Obes..

[6] Drożdżyńska J., Jakubowska W., Kemuś M., Krokowska M., Karpezo K., Wiśniewska M., Bogdański P., Skrypnik D. (2022). SARS-CoV-2 and Influenza Vaccines in People with Excessive Body Mass-A Narrative Review. Life.

[7] Andrade F.B., Gualberto A., Rezende C., Percegoni N., Gameiro J., Hottz E.D. (2021). The Weight of Obesity in Immunity from Influenza to COVID-19. Front. Cell. Infect. Microbiol..

[8] Dhurandhar N.V., Bailey D., Thomas D. (2015). Interaction of obesity and infections. Obes. Rev..

[9] Huttunen R., Syrjänen J. (2013). Obesity and the risk and outcome of infection. Int. J. Obes..

[10] Dobner J., Kaser S. (2018). Body mass index and the risk of infection—From underweight to obesity. Clin. Microbiol. Infect..

[11] Pellini R., Venuti A., Pimpinelli F., Abril E., Blandino G., Campo F., Conti L., De Virgilio A., De Marco F., Di Domenico E.G. (2021). Initial observations on age, gender, BMI and hypertension in antibody responses to SARS-CoV-2 BNT162b2 vaccine. eClinicalMedicine.

[12] Dalamaga M., Christodoulatos G.S., Karampela I., Vallianou N., Apovian C.M. (2021). Understanding the Co-Epidemic of Obesity and COVID-19: Current Evidence, Comparison with Previous Epidemics, Mechanisms, and Preventive and Therapeutic Perspectives. Curr. Obes. Rep..

[13] Vimercati L., De Maria L., Quarato M., Caputi A., Gesualdo L., Migliore G., Cavone D., Sponselli S., Pipoli A., Inchingolo F. (2021). Association between Long COVID and Overweight/Obesity. J. Clin. Med..

[14] Golec M., Fronczek M., Zembala-John J., Chrapiec M., Konka A., Wystyrk K., Botor H., Brzoza Z., Kasperczyk S., Bułdak R.J. (2022). Early and Longitudinal Humoral Response to the SARS-CoV-2 mRNA BNT162b2 Vaccine in Healthcare Workers: Significance of BMI, Adipose Tissue and Muscle Mass on Long-Lasting Post-Vaccinal Immunity. Viruses.

[15] Hu J., Zhao M., Zhao Y., Dong W., Huang X., Zhang S. (2022). Increased body mass index linked to decreased neutralizing antibody titers of inactivated SARS-CoV-2 vaccine in healthcare workers. Obes. Sci. Pract..

[16] Kara Z., Akçin R., Demir A.N., Dinç H.Ö., Taşkın H.E., Kocazeybek B., Yumuk V.D. (2022). Antibody Response to SARS-CoV-2 Vaccines in People with Severe Obesity. Obes. Surg..

[17] Pellini R., Venuti A., Pimpinelli F., Abril E., Blandino G., Campo F., Conti L., De Virgilio A., De Marco F., Di Domenico E.G. (2021). Early Onset of SARS-CoV-2 Antibodies after First Dose of BNT162b2: Correlation with Age, Gender and BMI. Vaccines.

[18] van der Klaauw A.A., Horner E.C., Pereyra-Gerber P., Agrawal U., Foster W.S., Spencer S., Vergese B., Smith M., Henning E., Ramsay I.D. (2023). Accelerated waning of the humoral response to COVID-19 vaccines in obesity. Nat. Med..

[19] Violán C., Carrasco-Ribelles L.A., Collatuzzo G., Ditano G., Abedini M., Janke C., Reinkemeyer C., Giang L.T.T., Liviero F., Scapellato M.L. (2023). Multimorbidity and Serological Response to SARS-CoV-2 Nine Months after 1st Vaccine Dose: European Cohort of Healthcare Workers-Orchestra Project. Vaccines.

[20] Golec M., Konka A., Fronczek M., Zembala-John J., Chrapiec M., Wystyrk K., Kasperczyk S., Brzoza Z., Bułdak R.J. (2022). The Antibody Response to the BNT162b2 mRNA COVID-19 Booster in Healthcare Workers: Association between the IgG Antibody Titers and Anthropometric and Body Composition Parameters. Vaccines.

[21] Lee S.W., Moon J.-Y., Lee S.-K., Lee H., Moon S., Chung S.J., Yeo Y., Park T.S., Park D.W., Kim T.-H. (2021). Anti-SARS-CoV-2 Spike Protein RBD Antibody Levels After Receiving a Second Dose of ChAdOx1 nCov-19 (AZD1222) Vaccine in Healthcare Workers: Lack of Association With Age, Sex, Obesity, and Adverse Reactions. Front. Immunol..

[22] Nasr M.-J.C., Geerling E., Pinto A.K. (2022). Impact of Obesity on Vaccination to SARS-CoV-2. Front. Endocrinol..

[23] Papaioannidou P., Skoumpa K., Bostanitis C., Michailidou M., Stergiopoulou T., Bostanitis I., Tsalidou M. (2023). Age, Sex and BMI Relations with Anti-SARS-CoV-2-Spike IgG Antibodies after BNT162b2 COVID-19 Vaccine in Health Care Workers in Northern Greece. Microorganisms.

[24] Watanabe M., Balena A., Tuccinardi D., Tozzi R., Risi R., Masi D., Caputi A., Rossetti R., Spoltore M.E., Filippi V. (2022). Central obesity, smoking habit, and hypertension are associated with lower antibody titres in response to COVID-19 mRNA vaccine. Diabetes Metab. Res. Rev..

[25] Yamamoto S., Mizoue T., Tanaka A., Oshiro Y., Inamura N., Konishi M., Ozeki M., Miyo K., Sugiura W., Sugiyama H. (2022). Sex-associated differences between BMI and SARS-CoV-2 antibody titers following the BNT162b2 vaccine. Obesity.

[26] Cegolon L., Mauro M., Sansone D., Tassinari A., Gobba F.M., Modenese A., Casolari L., Liviero F., Pavanello S., Scapellato M.L. (2023). A Multi-Center Study Investigating Long COVID-19 in Healthcare Workers from North-Eastern Italy: Prevalence, Risk Factors and the Impact of Pre-Existing Humoral Immunity-ORCHESTRA Project. Vaccines.

[27] WHO A Healthy Lifestyle—WHO Recommendations. https://www.who.int/europe/news-room/fact-sheets/item/a-healthy-lifestyle---who-recommendations.

[28] d’Errico M., Pavlova M., Spandonaro F. (2022). The economic burden of obesity in Italy: A cost-of-illness study. Eur. J. Health Econ..

[29] Li C.Y., Sung F.C. (1999). A review of the healthy worker effect in occupational epidemiology. Occup. Med..

[30] Porru S., Monaco M.G.L., Spiteri G., Carta A., Caliskan G., Violán C., Torán-Monserrat P., Vimercati L., Tafuri S., Boffetta P. (2023). Incidence and Determinants of Symptomatic and Asymptomatic SARS-CoV-2 Breakthrough Infections After Booster Dose in a Large European Multicentric Cohort of Health Workers-ORCHESTRA Project. J. Epidemiol. Glob. Health.

[31] Modenese A., Casolari L., Rossi G., Della Vecchia E., Glieca F., D’Elia C., Garavini D., Righi E., Mariani S., Venturelli L. (2021). Factors Associated with SARS-CoV-2 Infection Risk among Healthcare Workers of an Italian University Hospital. Healthcare.

[32] Rizza S., Coppeta L., Grelli S., Ferrazza G., Chiocchi M., Vanni G., Bonomo O.C., Bellia A., Andreoni M., Magrini A. (2021). High body mass index and night shift work are associated with COVID-19 in health care workers. J. Endocrinol. Investig..

[33] Popkin B.M., Du S., Green W.D., Beck M.A., Algaith T., Herbst C.H., Alsukait R.F., Alluhidan M., Alazemi N., Shekar M. (2020). Individuals with obesity and COVID-19: A global perspective on the epidemiology and biological relationships. Obes. Rev..

[34] Alishaq M., Nafady-Hego H., Jeremijenko A., Al Ajmi J.A., Elgendy M., Vinoy S., Fareh S.B., Plaatjies J.V., Nooh M., Alanzi N. (2021). Risk factors for breakthrough SARS-CoV-2 infection in vaccinated healthcare workers. PLoS ONE.

[35] Akhter M., Roy S.K., Khair A., Karim M.R., Mojlish U.K.F.K., Ahmed M.U., Ali L. (2024). SARS-CoV-2 breakthrough infection and its covariates among healthcare providers of a hospital in Bangladesh during the omicron wave. Heliyon.

[36] Visci G., Zunarelli C., Mansour I., Porru S., De Palma G., Duval X., Monaco M.G.L., Spiteri G., Carta A., Lippi G. (2022). Serological response after SARS-CoV2 vaccination in healthcare workers: A multicenter study. La Med. Del Lav..

[37] Collatuzzo G., Visci G., Violante F.S., Porru S., Spiteri G., Monaco M.G.L., Fillon F.L., Negro C., Janke C., Castelletti N. (2022). Determinants of anti-S immune response at 6 months after COVID-19 vaccination in a multicentric European cohort of healthcare workers—ORCHESTRA project. Front. Immunol..

