# Supplementary material for: Body Mass Index and COVID-19: An Overview Among an Italian Multicentric Cohort of Healthcare Workers in the Pre- and Post-Vaccination Eras—ORCHESTRA Project
Source: Vaccines (Basel). 2025 Jun 19;13(6):660. doi: 10.3390/vaccines13060660 (PMC12197647; doi:10.3390/vaccines13060660)
Supplement: Supplementary file 1 [file vaccines-13-00660-s001.zip › vaccines-3673430-supplementary.pdf]

Supplementary table

Supplementary table S1. Acute and post-acute symptom severity by BMI category.

Infection by Vaccination Status*		BMI				*p-value*
		**Normoweight n=3428**	**Underweight n=223**	**Overweight n=1282**	**Obese n=537**	
**Before vaccination**	**Acute symptoms severity**					
	No symptoms	55 (20.1)	1 (5.6)	22 (18.6)	7 (11.3)	0.216
	Minor symptoms	131 (48.0)	10 (55.6)	51 (43.2)	26 (41.9)	
	Major symptoms	87 (31.9)	7 (38.8)	45 (38.2)	29 (46.8)	
	**Post-acute severity symptoms**					
	No symptoms	124 (46.8)	4 (23.5)	51 (44.7)	18 (31.6)	0.143
	Minor symptoms	108 (40.7)	9 (53.0)	42 (36.8)	28 (49.1)	
	Major symptoms	33 (12.5)	4 (23.5)	21 (18.5)	11 (19.3)	
**After 3rd dose**	**Acute symptoms severity**					
	No symptoms	114 (14.3)	3 (6.2)	35 (13.6)	7 (7.4)	0.197
	Minor symptoms	510 (64.1)	34 (70.8)	176 (68.2)	61 (64.9)	
	Major symptoms	172 (21.4)	11 (23.0)	47 (18.2)	26 (27.7)	
	**Post-acute severity symptoms**					
	No symptoms	406 (53.8)	22 (45.8)	127 (52.1)	34 (37.8)	0.044
	Minor symptoms	279 (37.0)	22 (45.8)	103 (42.2)	46 (51.1)	
	Major symptoms	70 (9.2)	4 (8.4)	14 (5.7)	10 (11.1)	

** Data not reported for Between 1st and 3rd due to not sufficient data.*
